# National Survey on Access, Use and Promotion of Rational Use of Medicines (PNAUM): household survey component methods

**DOI:** 10.1590/S1518-8787.2016050006156

**Published:** 2016-12-01

**Authors:** Sotero Serrate Mengue, Andréa Dâmaso Bertoldi, Alexandra Crispim Boing, Noemia Urruth Leão Tavares, Tatiane da Silva Dal Pizzol, Maria Auxiliadora Oliveira, Paulo Sérgio Dourado Arrais, Luiz Roberto Ramos, Mareni Rocha Farias, Vera Lucia Luiza, Regina Tomie Ivata Bernal, Aluísio Jardim Dornellas de Barros

**Affiliations:** I Programa de Pós-Graduação em Epidemiologia. Faculdade de Medicina. Universidade Federal do Rio Grande do Sul. Porto Alegre, RS, Brasil; II Departamento de Medicina Social. Faculdade de Medicina. Universidade Federal de Pelotas. Pelotas, RS, Brasil; III Departamento de Saúde Pública. Centro de Ciências da Saúde. Universidade Federal de Santa Catarina. Florianópolis, SC, Brasil; IV Departamento de Farmácia. Faculdade de Ciências da Saúde. Universidade de Brasília. Brasília, DF, Brasil; V Departamento de Produção e Controle de Medicamentos. Faculdade de Farmácia. Universidade Federal do Rio Grande do Sul. Porto Alegre, RS, Brasil; VI Departamento de Política de Medicamentos e Assistência Farmacêutica. Escola Nacional de Saúde Pública Sérgio Arouca. Fundação Oswaldo Cruz. Rio de Janeiro, RJ, Brasil; VII Departamento de Farmácia. Faculdade de Farmácia, Odontologia e Enfermagem. Universidade Federal do Ceará. Fortaleza, CE, Brasil; VIII Departamento de Medicina Preventiva. Escola Paulista de Medicina. Universidade Federal de São Paulo. São Paulo, SP, Brasil; IX Departamento de Ciências Farmacêuticas. Centro de Ciências da Saúde. Universidade Federal de Santa Catarina. Florianópolis, SC, Brasil; X Núcleo de Pesquisas Epidemiológicas em Nutrição e Saúde. Departamento de Nutrição. Faculdade de Saúde Pública. Universidade de São Paulo. São Paulo, SP, Brasil

**Keywords:** Drug Utilization, statistics & numerical data, Pharmaceutical Services, supply & distribution, Sampling Studies, Health Surveys, methods

## Abstract

**OBJECTIVE:**

To describe methodological aspects of the household survey National Survey on Access, Use and Promotion of Rational Use of Medicines (PNAUM) related to sampling design and implementation, the actual obtained sample, instruments and fieldwork.

**METHODS:**

A cross-sectional, population-based study with probability sampling in three stages of the population living in households located in Brazilian urban areas. Fieldwork was carried out between September 2013 and February 2014. The data collection instrument included questions related to: information about households, residents and respondents; chronic diseases and medicines used; use of health services; acute diseases and events treated with drugs; use of contraceptives; use of pharmacy services; behaviors that may affect drug use; package inserts and packaging; lifestyle and health insurance.

**RESULTS:**

In total, 41,433 interviews were carried out in 20,404 households and 576 urban clusters corresponding to 586 census tracts distributed in the five Brazilian regions, according to eight domains defined by age and gender.

**CONCLUSIONS:**

The results of the survey may be used as a baseline for future studies aiming to assess the impact of government action on drug access and use. For local studies using a compatible method, PNAUM may serve as a reference point to evaluate variations in space and population. With a comprehensive evaluation of drug-related aspects, PNAUM is a major source of data for a variety of analyses to be carried out both at academic and government level.

## RESUMO

**OBJETIVO:**

Descrever aspectos metodológicos do inquérito domiciliar da Pesquisa Nacional sobre Acesso, Utilização e Promoção do Uso Racional de Medicamentos (PNAUM) quanto ao desenho e implementação da amostragem e da amostra efetivamente obtida, seus instrumentos e implementação do campo.

**MÉTODOS:**

Estudo transversal de base populacional com amostra probabilística em três estágios da população residente nos domicílios localizados na zona urbana do Brasil. O trabalho de campo foi desenvolvido entre setembro de 2013 e fevereiro de 2014. O instrumento de coleta de dados incluiu questões relativas a: informações do domicílio, dos moradores e dos entrevistados; doenças crônicas e medicamentos utilizados; uso de serviços de saúde; doenças e eventos agudos tratados com medicamentos; uso de contraceptivos; uso de serviços de farmácia; comportamentos que podem afetar o uso de medicamentos; bulas e embalagens; estilo de vida e planos de saúde.

**RESULTADOS:**

Foram realizadas 41.433 entrevistas em 20.404 domicílios e 576 conglomerados que correspondem a 586 setores censitários distribuídos nas cinco regiões do Brasil, segundo oito domínios definidos por grupos de idade e sexo.

**CONCLUSÕES:**

Os resultados obtidos no inquérito podem ser utilizados como uma linha de base para futuros estudos que pretendam avaliar o impacto de ações governamentais nas áreas de acesso e de utilização de medicamentos. Para estudos locais que venham a usar um método compatível, a PNAUM pode servir como ponto de referência para avaliação de variações do espaço e da população. Com ampla avaliação dos aspectos relacionados aos medicamentos, a PNAUM é uma grande fonte de dados para as mais variadas análises, que podem ser conduzidas tanto no meio acadêmico como no âmbito governamental.

## INTRODUCTION

Government investment in health has increased in recent years. Brazilian Ministry of Health (MH) expenditure with medicines has accompanied this growth, ranging from R$1.8 billion to R$12.4 billion between 2003 and 2014, while maintaining the average budget ratio of about 14.0%^4^. Adjusting for inflation over the period, there was a 3.6 times increase in real value. Despite increased spending on medicines, no assessment of its impact on drug access and use by the Brazilian population was heretofore available.

Strategies to evaluate drug access policies involve various aspects, ranging from macroeconomic issues to the obtainment and use of these drugs by the population. Evaluation of drug use and access in several countries has been based on models of drug supply, which can be via free provision, health insurance coverage, refund or direct payment^a^.

National health studies in Brazil date back to 1981, with the first health supplement of the *Pesquisa Nacional de Amostra por Domicílio* (PNAD – National Household Sample Survey), repeated in 1986, 1998, 2003 and 2008^b^ ^,^ ^c^. The various PNAD editions were limited to surveying medicines of long-term use and their forms of access. In the 2013 *Pesquisa Nacional de Saúde* (PNS – National Survey on Health)^d^, information on obtaining medicines is equivalent to that of PNAD, but with separate information for diabetes and high blood pressure^14^.

The biggest limitation to evaluating drug use in health surveys has been the level of detail. Besides the fact that the surveys must address a broad number of topics, measuring drug use is complex and involves many difficulties, from recording drug names correctly to questions on the various reasons for using medicines and how they are obtained.

Given this situation, the Brazilian Ministry of Health proposed in 2009 the development of the first national survey on drug access and use: *Pesquisa Nacional Sobre Acesso, Utilização e Promoção do Uso Racional de Medicamentos* (PNAUM – National Survey on Access, Use and Promotion of Rational Use of Medicines). This survey was established by ordinance GM/MS 2077 from September 17, 2012 to address the need of information related to drug access, use and rational use in Brazil, in accordance with MH strategic objectives, which include ensuring pharmaceutical services and reducing inadequate access to health and pharmaceutical services provided by the Brazilian Unified Health System (SUS)^e^.

PNAUM was organized into two components: 1) household survey on drug access, use and rational use, the focus of this article, and 2) evaluation of public policies for pharmaceutical services and their implementation in SUS Primary Care Health. The PNAUM household survey aimed to evaluate the use of medicines by the Brazilian population, characterizing the morbidities for which they are used, access indicators and rational use of drugs according to demographic, socioeconomic, lifestyle and morbidity variables.

This article aims to describe methodological aspects of the household survey related to sampling design and implementation, instruments and fieldwork, as well as the sample obtained.

## METHODS

PNAUM is a cross-sectional, population-based study with probability sampling, carried out between September 2013 and February 2014 in urban households at national level. The survey was restricted to the urban population due to logistics difficulties and the additional cost involved in studying rural areas.

### Sample Design

The medicine use varies according to age and gender regarding the amount and type of medication, and it is essential to ensure accurate estimates of both groups. Thus, the sample included eight demographic domains: (1) ages 0-4, both genders; (2) ages 5-19, both genders; (3) ages 20-39, female; (4) ages 20-39, male; (5) ages 40-59, female; (6) ages 40-59, male; (7) ages 60 or over, female; (8) ages 60 or over, male.

The demographic domains were replicated for each of the major Brazilian geographical regions, resulting in 40 sampling domains, ensuring the accuracy of indicators of interest for the subgroups selected within each region. Sample size was defined based on estimates obtained from the 2008 PNAD. These estimates were: 34.0% of totally free access to medications, 44.0% of paid access and the remainder of mixed access. Also used was a 12.0% estimate of lack of access to needed medicines. The precision criterion was set at 0.05, the maximum value for variation coefficients from the estimates of interest. This resulted in a minimum sample size of 960 interviews per sampling domain, totaling 38,400 interviews.

The sample was drawn from clusters in three stages: municipality (primary sampling unit), census tract and household. The municipalities were selected by systematic sampling with probability proportional to size within each region, totaling 60 clusters for each one of them. Two tracts within each selected municipality were also selected with probability proportional to size. In the third stage, 86, 72, 70, 54 and 61 households were drawn in the North, Northeast, Midwest, Southeast and South regions, respectively. This total is the number of households increased by 10.0% due to refusal. The household draw used the 2010 *Cadastro Nacional de Endereços do Censo* (National Census Address Register) of the Brazilian Institute of Geography and Statistics (IBGE). Before the fieldwork, the tracts were visited for a quick household count to detect changes. Tracts with less than 90.0% of update for valid addresses were updated immediately prior to the interview and selection process. The household draw in each domain was based on the expected percentage for each age and gender group. The number of individuals in each group was previously defined in order to compose the sample with the desired numbers. In the sample selection process, 2010 Census data were used as a source of the number of households and individuals^f^.

Sampling weights were calculated for individuals, and post-stratification weights were used to reduce the bias resulting from low response rate. These weights were calculated by the raking method, using the population distribution estimated by the PNS according to age and gender per region^g^.

The following categories were used to calculate response rates:

1D - actual sample (household with eligible resident);

2D - incompatible profiles (household without eligible resident);

3D - inexistent, not located or inaccessible household;

4D - closed or abandoned household;

5D - refusal (to report about eligible population in household);

6D - non-residential;

7D - not visited (difference between number of addresses predicted and visited).

The response rate for households (TR_D_) was calculated by:


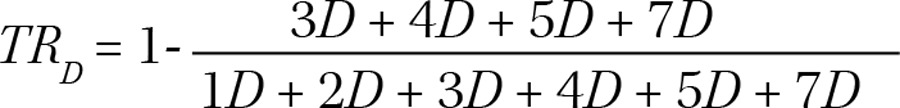


Non-residential households were excluded from the calculation, as vacant households should also have been. The latter were not excluded for lack of information about them. Closed households (considered non-responsive) and abandoned households were included in a single category.

For residents, the response rate (TRM) was calculated by:


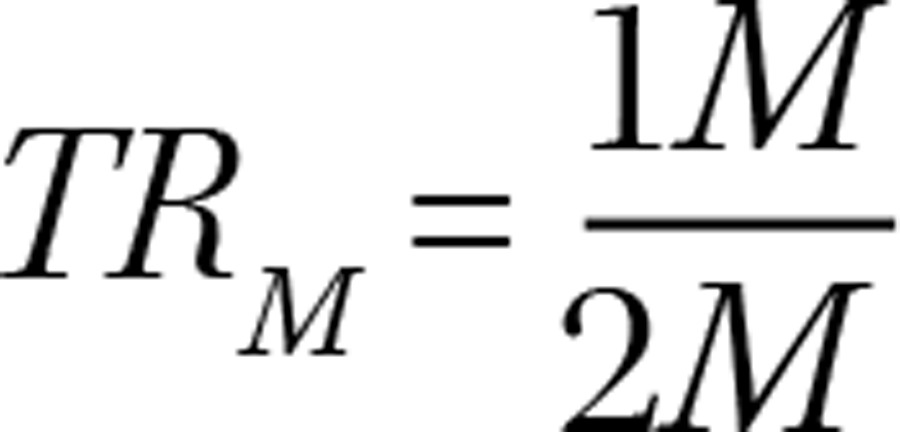


1M - Interviewed resident

2M - Eligible resident

Based on the DHS (Demographic and Health Surveys - Phase III^h^) sampling manual, the following was used as overall response rate:


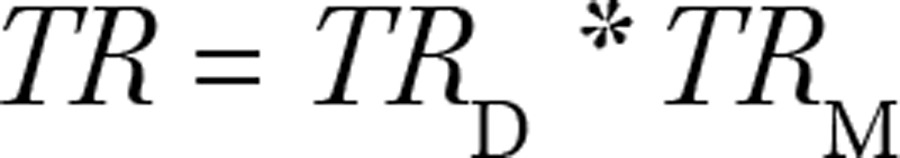


In some demographic domains by region, the originally planned sample size was not obtained. Thus, a post-stratification process was carried out by applying to the sample observations the weights related to the new sampling fractions adjusted by region, gender and age, following the Brazilian population distribution from the 2013 PNS. This procedure ensured a sample distribution consistent with the distribution of the Brazilian population.

### Survey Instruments

A questionnaire for adults was developed with 11 blocks. This questionnaire was adapted to be answered by the person responsible for the care of children and incapable people (Table 1). Full versions of the questionnaires used are available on the PNAUM website (http://www.ufrgs.br/pnaum).

**Table 1:** Breakdown of questionnaires administered to individuals aged > 15, incapable or aged < 14, by blocks. PNAUM, Brazil, 2104.

Block		Section	Aged 15 or over	Incapable of answering interview	Aged below 15
1	Respondent information	Gender Age Level of education Marital status Skin color/ethnicity Self-reported height and weight	✓	Data of substitute respondent: gender, age, level of education and kinship to participant	Data of substitute respondent: gender, age, level of education and kinship to participant
2	Chronic diseases	High blood pressure Diabetes Heart diseases Hypercholesterolemia Stroke Pulmonary disease Arthritis, arthrosis or rheumatism Depression Other chronic diseases lasting 6 months or longer	✓	✓	Respiratory diseases Diabetes Other chronic diseases lasting 6 months or longer
2.1	List of medications for chronic diseases	Detailed description of medications used for chronic diseases	✓	✓	✓
3	Health Services	Use of health services Information on medical care	✓	✓	✓
4	Acute diseases or events treated with medicines	Infection Sleeping or anxiety problems Stomach or bowel problems Flu Cold or rhinitis Fever and pain Use of vitamins, mineral supplements, appetite stimulants or tonics	✓	✓	Nausea, vomiting and diarrhea were included and sleeping or anxiety problems were excluded
4.1	List of medications for acute diseases and events	Detailed description of medications used for acute diseases and events	✓	✓	✓
5	Contraceptives	Oral or injectable contraceptives	✓	✓	✓
6	Pharmacy services	SUS pharmacy Private sector pharmacy Popular pharmacy	✓	✓	✓
7	Behaviors that may affect drug use	People or other sources of reference for indication of drugs Sources of information on drugs Behaviors related to self-medication and non-adherence Guidance on drug storage Guidance on drug use length of time	✓		
8	Inserts and packaging	Use of inserts Understanding of inserts	✓		
9	Lifestyle	Physical activity Smoking Alcohol consumption	✓		
10	Health insurance	Health insurance Insurance coverage	✓	✓	✓
11	Household and head of household information	Household information Head of household information	✓	✓	✓

TCLE: Informed consent

Before the actual interview, the name, gender and age of all household residents were recorded. This information was used to identify the domains to be interviewed at that household.

For “children” (persons below 15 years of age), blocks 2 and 4 were adapted and blocks 5, 7, 8 and 9 were not used. Blocks 5, 7, 8 and 9 were not used for incapable people either, defined here as those unable to communicate or self-report information due to physical or mental illness, speech impediment or lack of discernment to answer questions.

The first block features respondent data related to the three groups of people. The second investigates chronic diseases and the current use of drugs in each disease. The selection of the reasons for drug use was based on the most prevalent health problems in the population and the 2008 PNAD Health assessment (IBGE)^i^. Respondents were asked about the existence of medical diagnosis: high blood pressure, diabetes, heart diseases, hypercholesterolemia, stroke, lung disease, arthritis, arthrosis or rheumatism, depression and other chronic diseases lasting six months or longer. For each medication, detailed information was collected about the product (generic, expiry date, dosage form, concentration) and its use (length of time, dosage, source, among other information). To identify adherence barriers related to therapy, beliefs and memories regarding the long-term use of medications, the Brief Medication Questionnaire (BMQ)^1^ was used.

The third block investigates the types of health services used. Block 4 contains questions about the use of drugs in the previous 15 days for acute events or diseases. For each medicine, a set of questions was asked to obtain detailed information.

To record data on drugs reported in blocks 2 and 4, respondents were asked to show all “medicines” being use. Any product used to cure or alleviate diseases, symptoms, discomfort or mild illness was considered medicine. Thus, a medicine could be a compounded or processed drug or herbal tea, homeopathic product or medicinal plant, for example.

Block 5 investigates the current use and access to oral and injectable contraceptives, including means of obtainment and name of product, as well as data on side effects and treatment adherence among women aged 15 to 49. The questions were adapted from the 2006 *Pesquisa Nacional de Demografia e Saúde* (National Demographics and Health Survey)^j^.

Block 6 was designed to identify in detail the sources for obtaining different drugs, given that individuals may use various services to obtain all medications needed. Block 7 features questions to evaluate behaviors that may affect drug use. Block 8 investigates respondents’ habits regarding the reading of the package inserts and drug storage, containing questions adapted from Didonet and Mengue (2008) and Silva et al. (2000)^12^.

Block 9 contains questions related to the use of tobacco and alcohol, based on the 2011 Vigitel^k^, and to physical activity based on the Global Physical Activity Questionnaire – GPAC^l^. Block 10 asks whether the respondent has health insurance or not and the items covered, especially medications.

The last block, consisting of two sections, was used to collect household information. The first section gathered information such as household goods, furniture, household income and number of rooms in order to apply the *Critério de Classificação Econômica Brazil* (CCEB – Brazilian Economic Classification Criterion) of *Associação Brasileira das Empresas de Pesquisa* (ABEP – Brazilian Association of Survey Companies). The second section gathered sociodemographic information of the head of household.

The interview flowchart according to the order of blocks is described in the Figure.

**Figure:**
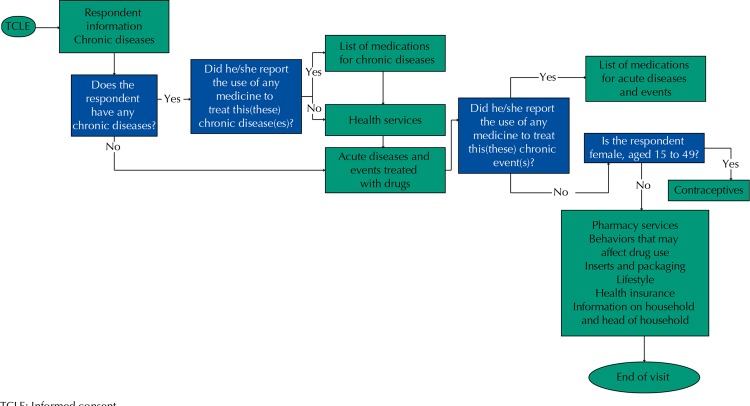
Interview flowchart. The upper flowchart refers to interviews with adults and the bottom to interviews with incapable people and children. PNAUM, Brazil, 2014.

### Fieldwork

#### Team and Structure

The field team was formed by coordination core, operational support team, and interviewers. The coordination core was responsible for overseeing all collection processes and stages. The operational support team carried out field supervision and provided logistics and administrative support to the teams gathering data. A support center was also set up to clarify doubts and solve operational problems, working full time throughout the research.

Two-hundred and seventeen potential interviewers were trained, 165 of whom took part in the actual data collection. A three-day training was given by the operational support team and PNAUM researchers. The training was organized into six different groups according to the Brazilian region where the interviews were done.

#### Pilot

To test field logistics, six pilot studies were carried out, one in each state capital where the training took place, totaling 251 interviews. Items tested at this point included the data collection instrument, support manual, data recording software, tablet operation and data transmission used in the fieldwork stage.

#### Survey Disclosure Strategies

Knowledge of the study by the population and institutional support of health and social assistance departments could facilitate interviewers’ contact with respondents and increase participation. Thus, brochures, posters and letters explaining the survey were distributed at the locations drawn for data collection, and the Health Hotline (136) was used for the population to confirm the veracity of the survey.

#### Data Collection

Having the required number of households per census tract was essential for the household draw. Thus, 124 census tracts required previous inventory due to the lack of updated information on the total number of households.

In households with eligible age groups, the interviewer would identify all residents through the person available at the first contact and carry out the interview with individuals from the selected age group. The household questions were answered by the head of household, while those related to the other blocks were answered individually by the respondents. In the case of children under 15 and incapable persons, the interview was conducted with the person named as responsible for their medication.

#### Quality Control

The quality control of the interviews was carried out through re-interviews with part of the sample, regular analysis during the survey of the frequency of investigated variables, and analysis of database consistency.

The re-interviews were conducted through telephone calls, using a standardized questionnaire with drawn respondents. This stage involved 5.123 re-interviews, corresponding to 12.0% of the sample. The reproducibility of variables was tested from the answers, and Kappa values were obtained showing a high level of agreement, ranging from 0.723 to 0.879.

Moreover, variable frequencies were monitored throughout the whole fieldwork, related to sociodemographic data, self-reported diseases, health service use, drug use, lifestyle, health insurance and assets. The values obtained were routinely compared with the most recent results of national surveys.

#### Ethical Aspects

PNAUM was approved by *Comissão Nacional de Ética em Pesquisa* (National Research Ethics Commission – Protocol 18947013.6.0000.0008) and *Comitê de Ética em Pesquisa da Universidade Federal do Rio Grande do Sul* (Research Ethics Committee of the Federal University of Rio Grande do Sul – Protocol 19997). All interviews were conducted after the respondents or their legal representatives (in the case of incapable persons) had read and signed the informed consent, with assurance of confidentiality and anonymity.

## RESULTS

### Response Rates

Response rates for households were between 42.0% and 60.0%, including as losses those households that, despite featuring in the draw list, were not visited because their residents could not be contacted. Among those are temporarily or permanently vacant households, since the survey kept no specific record for non-visited households. Response rates for individuals were between 82.0% and 97.0%. Overall response rates ranged from 46.6% to 56.1%. Due to the inclusion of vacant homes among losses, response rate values are somewhat underestimated. Table 2 features response rates by demographic domains and region.

**Table 2:** Households not visited and response rates, by region and age and gender domain. PNAUM, Brazil, 2014.

Region/Age and gender domain		% households not visited	Response rate/household	Response rate/individual	Overall response rate
Region	North	12.4	58.8	95.3	56.1
	Northeast	17.5	53.6	95.3	50.1
	Southeast	10.1	58.1	91.2	53.0
	South	17.6	53.5	93.1	49.8
	Midwest	34.4	42.4	91.5	38.8
Domain: (age group, gender)	0 to 4	18.1	52.8	95.2	50.2
	5 to 19	16.6	60.5	82.8	50.1
	20 to 39, male	16.7	58.8	84.0	49.3
	20 to 39, female	19.6	55.1	96.9	53.4
	40 to 59, male	19.1	55.1	93.6	51.6
	40 to 59, female	18.9	54.1	96.7	52.3
	≥ 60, male	18.1	51.7	90.1	46.6
	≥ 60, female	18.1	51.5	93.7	48.3

A negative correlation was clear between average income of the tract and overall response rate, albeit low, ranging from -0.39 to -0.02 (Table 3). Statistically significant correlations were observed between the different regions, age groups and genders, especially between adults and older adults.

**Table 3:** Correlation between average income of tract and overall response rate per domain according to region. PNAUM, Brazil, 2014.

Domain (age group, gender)	Measurements	Region				

		North	Northeast	Southeast	South	Midwest
0 to 4	Correlation	-0.14	-0.21	-0.17	-0.23	-0.29
	p	0.13	0.03	0.09	0.02	< 0.01
5 to 19	Correlation	-0.02	-0.10	-0.06	−0.24	-0.33
	p	0.86	0.31	0.58	0.02	< 0.01
20 to 39, male	Correlation	-0.10	-0.09	-0.23	-0.28	-0.16
	p	0.31	0.40	0.02	0.01	0.17
20 to 39, female	Correlation	-0.21	-0.28	-0.20	-0.36	-0.29
	p	0.02	< 0.01	0.04	< 0.01	< 0.01
40 to 59, male	Correlation	-0.14	-0.28	-0.18	-0.31	-0.26
	p	0.13	< 0.01	0.05	< 0.01	0.01
40 to 59, female	Correlation	-0.21	-0.32	-0.30	-0.24	-0.22
	p	0.02	< 0.01	< 0.01	0.01	0.03
60 or over, male	Correlation	-0.19	-0.32	-0.19	-0.22	-0.35
	p	0.04	< 0.01	0.05	0.02	< 0.01
60 or over, female	Correlation	-0.18	-0.32	-0.25	-0.39	-0.34
	p	0.05	< 0.01	0.01	< 0.01	< 0.01

### Sample Description

The survey interviewed 41,433 people who, following adjustments by region, gender and age, represent approximately 171 million Brazilians living in urban areas of the country. The interviews were conducted in 20,404 households and 576 clusters corresponding to 586 census tracts (10 census tracts were joined to others due to their small size). The numbers of interviews by region and demographic domain are featured in Table 4.

**Table 4:** Number of interviews made by region and demographic domain. PNAUM, Brazil, 2014.

Region	Domain*								Total
	Age (years) and gender								

	0-4	5-19	20-39	20-39	40-59	40-59	≥ 60	≥ 60	
	MF	MF	M	F	M	F	M	F	
North	1.835	667	552	2.538	1.539	1.778	899	1.115	10.923
Northeast	1.267	618	454	1.881	991	1.521	794	1.268	8.794
Southeast	817	606	533	1.363	1.059	1.267	728	1.125	7.498
South	872	877	562	1.365	1.078	1.299	784	1.009	7.846
Midwest	989	537	336	1.281	839	1.093	554	743	6.372

Total	5.780	3.305	2.437	8.428	5.506	6.958	3.759	5.260	

* The domains refer to the age and gender groups (M = male; F = female; MF = male and female).

Table 5 features the general characteristics of the sampled population, according to age group, gender, self-reported skin color, level of education, marital status, economic class and geographical region. The Southeast region represented 45.9% of the sample; Northeast, 24.3%; South, 14.3%; Midwest, 7.9%; and North, 7.5%. There was a slight predominance of females (52.8%); 31.9% were aged 20-39; and 46.0% self-reported as white. Blacks and mulattoes combined are the majority among the population (52.5%). Economic bracket C was predominant, accounting for 55.3% of respondents. Of individuals aged 20 or older, 36.1% reported zero to three years of study.

**Table 5:** Sample breakdown by demographic and socioeconomic characteristics. PNAUM, Brazil, 2014. (N = 41,433)

Variable	Category	%^a^	95%CI
Age group (years)	0-4	6.2	5.9–6.5
	5-10	9.1	8.3–9.8
	11-19	14.4	13.3–15.5
	20-39	31.9	30.4–33.3
	40-59	25.3	24.3–26.4
	≥ 60	13.2	12.4–14.0
Gender	Male	47.2	46.3–48.1
	Female	52.8	51.9–53.7
Skin color or ethnicity (self-reported)	White	46.0	43.5–48.6
	Black	8.7	7.9–9.7
	Mulatto	43.8	41.5–46.1
	Yellow	1.1	0.9–1.3
	Indigenous	0.4	0.3–0.5
Level of education^b^	0-3 years	36.1	34.5–37.7
	4-11 years	31.9	30.8–33.0
	≥ 12 years	32.0	30.6–33.5
Marital status^b^	Partner	60.7	59.4–62.1
	No partner	39.3	37.9–40.6
CCEB^c^	A	0.5	0.3–0.9
	B	21.9	20.0–23.9
	C	55.3	53.7–57.0
	D	17.7	16.1–19.5
	E	4.6	4.0–5.3
Region	North	7.5	5.9–9.5
	Northeast	24.3	20.1–29.1
	Southeast	45.9	40.0–52.0
	South	14.3	11.5–17.8
	Midwest	7.9	6.2–10.0

^a^ Percentage adjusted for sampling weights and post-stratification according to age and gender.

^b^ Only adults aged 20 or over.

^c^ Classified according to *Critério de Classificação Econômica Brasil* 2013 (CCEB 2013 – Brazilian Economic Classification Criterion) of *Associação Brasileira de Empresas de Pesquisa* (ABEP – Brazilian Association of Survey Companies). Available from: http://www.abep.org

## DISCUSSION

PNAUM is the first population-based survey on drug access and use carried out in Brazil with national representation. Previous studies in the area were conducted using different methods, diverse target populations and local scope, which hindered comparison of results and limited the generalization of findings to the Brazilian population. The information obtained in this study enables the assessment of drug use profiles and the impact of Brazilian drug policies on access to medicines.

Studies on drug use can be divided into two groups. The first performs secondary analyses of databases of direct or refunded payments for medicines. In such cases, the analyses do not address the actual use of drugs nor those acquired outside the health system. These are the most used studies in developed countries with integrated information systems. The second group performs household surveys, face-to-face or by phone. These are primarily used in countries that do not have such information systems – generally poorer countries.

In general, the surveys have a restricted approach to individual drug use, either limited to a group of diseases, with questions such as “use or not,” or providing a list of medicines from which the user is asked to give information^11^. Others, such as the Australian Health Study, ask for a list of medicines and investigate use for specific diseases^m^.

More detailed, nationwide studies have been conducted in the United States and include the Slone Survey (2004 to 2006) and the Medical Expenditure Panel Survey (MEPS) that, since 1996, collects data on prescription and over-the-counter drugs^n^.

In countries of Africa, Asia, Latin America and the Caribbean, studies on drug access and use have been performed using the methodology proposed by the World Health Organization and also adopted by the Medicines Transparency Alliance (MeTA)^8^. The model addresses a comprehensive assessment of drug policy in countries, ranging from government aspects through public and private services to household surveys^3^.

Some of the methodological challenges and constraints identified in this study are discussed below.

Comparisons with other national household studies show that response rates are lower than those obtained by the IBGE in the 2013 PNS, in which the response rate was 91.9% for households and 86.0% for individuals, resulting in an overall response rate of 79.0%. However, such comparisons are compromised, since the PNS estimate excluded vacant households. PNAUM, on the contrary, preserved vacant households in the calculation due to the lack of field records. The *Pesquisa Nacional de Saúde Bucal* ^o^ (National Oral Health Survey) obtained general response rates ranging from 27.6% in Cuiabá (MT) to 134.3% in Porto Alegre (RS). The study did not describe the overall response rate, but a rough estimate shows that, for people aged 35 to 44, the average overall response rate is 50.0%. For older adults and children, this value was above 80.0%, ranging from 55.0% to 100%.

Response rates in household surveys have shown a steady decline in the last 40 years almost all over the world. In the United States, the National Health Survey Interview showed a decrease in response rate from 92.0% in 1997 to 89.0% in 2004; the National Expenditure Panel Survey, from 78.0% in 1996 to 53.0% in 2014; and the Behavioral Risk Factor Surveillance System, from 71.4% in 1993 to 48.9% in 2000 and 51.1% in 2005^7^. Tolonen et al. observed a reduction in response rate in the Finnish Adult Health Behaviour Survey response rate from 80.0% in 1978 to 60.0% in 2002. In an evaluation of the results of surveys conducted in Europe between 2007 and 2012, Mindell et al.^10^ considered as good the response rates of 66.0% in England, 54.0% in Germany and 45.0% in the Netherlands.

In previous studies that discuss the reduction of response rates, a few reasons are most frequently suggested. The first is people’s reaction to a great number of surveys carried out in the most diverse areas, such as market, politics and health. Telephone surveys have particularly bothered people, since they are linked to marketing activities in general. A growing concern with privacy has influenced the decline in response rates. In PNAUM, part of the population worried greatly about safety, which significantly prevented access to residents of larger buildings. This kind of worry seems to be an additional component diminishing the receptivity of people to household surveys.

The quality of information obtained through interviews is a problem to be considered. Of used medicines, 39.0% had no packaging available. In such cases, drug identification is based solely on report, which requires comprehensive reviews and subsequent correction to data collection. Approximately 4.0% of all medicines used could not be identified. In addition to those, food supplements, or food reported as medicine, medicinal plants and cosmetics amounted to 4.1%. In some cases, even with the packaging in hand, respondents were not able to link the drugs to each one of the treated diseases. This generated inconsistent information between the declared disease and the therapeutic indication of the drug.

The correct identification of medicines is a common difficulty in household studies. Contraceptives or vitamins, e.g., are frequently omitted in spontaneous reports on medicines when they are not being used to treat any disease. On the other hand, food supplements and other products are often reported as drugs to treat diseases. It should be noted that some medications have what might be called a “compound name,” with the first word identifying the product and the second referring to a different composition, which results in another pharmaceutical product. At the same time, medicines may come in different concentrations, which may relate to different uses (such as case of acetylsalicylic acid 345 mg and 500 mg). Additionally, some fixed–dose combinations have different proportions in each dosage form. This demands hard work to identify and classify products following data collection to generate the necessary knowledge on the use of those therapeutic resources.

Moreover, respondents’ perception of the definition of chronic disease demands analysis to produce a classification that enables the best possible expression of the health conditions described by people^9^.

Correct identification of where the respondents obtained their medicines was a limitation in data collection. There are several ways of obtaining discount drugs, such as customer loyalty programs, which coexist, for example, with the program *Aqui tem Farmácia Popular* (Popular Pharmacy Here). At the same time, supply of medicines financed by SUS can occur in health facilities or public health pharmacies with mixed systems, in which part of the drugs is available in health facilities and part in centralized pharmacies.

The assessment of acute events examined the use of drugs to treat such diseases or their symptoms. Prevalence of these events, treated with non-pharmacological approaches or untreated, was not estimated.

The PNAUM instruments were developed in paper form. The blocks were first tested separately, with the complete tool assembled for final testing in a second stage. The electronic device version was developed later and reviewed during the pilot study. Transference of questionnaires to the small-screen tablet system revealed difficulties not observed in the hard copy versions and facilities available only to electronic equipment. This showed that, at least for this type of survey, electronic versions for testing should be used as soon as possible in the development of data collection instruments.

The use of these portable electronic devices in data collection also adds more stages to interviewer training. The first stage deals with the use of the actual equipment. The second covers the specifics of the software used in developing the application. The third stage is when the actual content training begins.

PNAUM fieldwork was carried out by a survey company hired specifically for this investigation. Working with contractors requires special care, especially regarding the schedule of each stage, proper sizing of field teams, monitoring progress and interviewing goals in each sample domain.

PNAUM is the first large national survey to face the challenge of investigating drugs in detail. Objective assessment of drug use requires identifying exactly which drugs are being used, which drugs are missing and why, where each product is obtained and how each product is being used. This information is needed to guide public programs, whether aimed at specific diseases or general coverage. In short, knowing what is being used, by whom, where and why is what guides the development of this initiative.

PNAUM may be used as a baseline for future studies aiming to assess the impact of government action on drug access and use. For local studies using a compatible method, PNAUM may serve as a reference point to evaluate variations in space and population. With a comprehensive evaluation of drug-related aspects, PNAUM is a major source of data for a variety of analyses to be carried out at both academic and government level.

## Footnotes

a Brasil, Conselho Nacional de Secretários de Saúde. O desafio do acesso aos medicamentos nos sistemas públicos de saúde: relatório do Seminário Internacional de Assistência Farmacêutica do CONASS. 2010 [cited 2015 Jan]. Available from: http://www.conass.org.br/conassdocumenta/cd_20.pdf

b Instituto Brasileiro de Geografia e Estatística. Pesquisa nacional por amostra de domicílios. Rio de Janeiro (RJ): IBGE; 2003.

c Instituto Brasileiro de Geografia e Estatística. Pesquisa nacional por amostra de domicílios. Rio de Janeiro (RJ): IBGE; 2008.

d Instituto Brasileiro de Geografia e Estatística. Pesquisa Nacional de Saúde 2013: percepção do estado de saúde, estilos de vida e doenças crônicas - Brasil, Grandes Regiões e Unidades da Federação. Rio de Janeiro (RJ): IBGE; 2014.

e Ministério da Saúde. Secretaria de Ciência, Tecnologia e Insumos Estratégicos, Departamento de Ciência e Tecnologia. Pesquisas estratégicas para o sistema de saúde - PESS. Brasília (DF): Ministério da Saúde; 2011.

f Instituto Brasileiro de Geografia e Estatística. Censo demográfico 2010: características da população e dos domicílios: resultado do universo. Rio de Janeiro (RJ): IBGE; 2011.

g Flores-Cervantes I, Brick JM; Jones ME. Weighting for nontelephone household in the 2001 California Health Interview Survey. In: Proceedings of the Joint Statistical Meetings Section on Survey Research Methods; 2002 [cited 2016 Aug 9] Aug 11-15; New York. p. 1002-7. Available from: http://www.amstat.org/sections/srms/Proceedings/y2002/Files/JSM2002-000661.pdf

h Macro International Inc. Demographic and health survey, phase III: sampling manual. Maryland: Macro International Inc; 1996 [cited 2016 Aug 9]. (DHS-III basic documentation, n6). Available from: http://dhsprogram.com/pubs/pdf/AISM5/DHS_III_Sampling_Manual.pdf

i Instituto Brasileiro de Geografia e Estatística. Pesquisa nacional por amostra de domicílios. Rio de Janeiro (RJ): IBGE; 2008.

j Ministério da Saúde. Centro Brasileiro de Análise e Planejamento. Pesquisa Nacional de Demografia e Saúde da Criança e da Mulher – PNDS 2006: dimensões do processo reprodutivo e da saúde da criança. Brasília (DF): Ministério da Saúde, 2009 [cited 2016 Aug 9]. Available from: http://bvsms.saude.gov.br/bvs/publicacoes/pnds_crianca_mulher.pdf

k Ministério da Saúde. Secretaria de Vigilância em Saúde. Secretaria de Gestão Estratégica e Participativa. Vigitel Brasil 2010: vigilância de fatores de risco e proteção para doenças crônicas por inquérito telefônico. Brasília (DF): Ministério da Saúde; 2011. p. 152.

l World Health Organization (WHO). Chronic diseases and health promotion: global physical activity surveillance. Geneva: World Health Organization; 2007 [cited 2016 Aug 9]. Available from: http://www.who.int/chp/steps/GPAQ/en/

m National Health Survey: Users’ Guide – Eletronic. Canberra: Australian Bureau of Statistics; 2009 [cited 2016 Aug 9]. Available from: http://www.ausstats.abs.gov.au/ausstats/subscriber.nsf/0/CC0FB5A08570984ECA25762E0017CF2B/$File/4363055001_2007-08.pdf

n Slone. Epidemiology Center. Slone Survey. Boston: Boston University; 2006 [cited 2016 Aug 9]. Available from: http://www.bu.edu/slone/research/studies/slone-survey/

o Ministério da Saúde. Secretaria de Atenção à Saúde. Secretaria de Vigilância em Saúde. SB Brasil 2010: Pesquisa Nacional de Saúde Bucal: resultados principais. Brasília (DF): Ministério da Saúde, 2012 [cited 2016 Aug 9]. Available from: http://bvsms.saude.gov.br/bvs/publicacoes/pesquisa_nacional_saude_bucal.pdf
